# Effects of Solid Solution Heat Treatment on the Corrosion Behavior of 800H Used in Fourth-Generation Nuclear Power Generators

**DOI:** 10.3390/ma19010143

**Published:** 2025-12-31

**Authors:** Yu Liu, Xiaoyuan Guo, Min Wang, Kaixing Yao, Huiqing Dong, Yafan Li, Zhidong Wang, Feng Wang, Rui Luo

**Affiliations:** 1 School of Materials Science & Engineering, Jiangsu University, Zhenjiang 212013, China; 13284620466@163.com (X.G.); w16606732729@163.com (M.W.); 19741706911@163.com (K.Y.); m19859536889@163.com (H.D.); lifeyfan@163.com (Y.L.); 2 Jiangsu Yinhuan Precision Steel Pipe Co., Ltd., Yixing 214200, China; wzdybyq@163.com (Z.W.); wfyx1988@163.com (F.W.); 3 Jiangsu Key Laboratory of Advanced Structural Materials and Application Technology, Nanjing Institute of Technology, Nanjing 211167, China

**Keywords:** 800H, corrosion resistant performance, heat treatment, microstructure

## Abstract

Incoloy 800H is important structural alloy for heat exchange tubes of Generation IV nuclear power systems. Revealing the key heat treatment effects on the microstructure and corrosion behavior of 800H is a key issue for its performance optimization and safe application in IV nuclear power industries. This work investigated the solid solution heat treatment–microstructure–corrosion resistance relationship using various electrochemical corrosion techniques and morphology characterizations. The results showed that 1120 °C was an insufficient solid solution heat treatment temperature for 800H, at which 800H demonstrated uneven enlargement of grains and undissolved Cr-carbides, which resulted in fast corrosion. 800H demonstrated even growth of grains with best grain uniformity and dissolution of Cr-carbides at 1150 °C, thus showing the best corrosion resistance. However, the further increase in solid solution temperature to 1180 °C resulted in excessive grain growth and severe intergranular corrosion (IGC) attack. This work deepened the understanding of the corrosion mechanism of 800H and provided data for its performance optimization.

## 1. Introduction

Nuclear energy, as a clean, safe, efficient, and stable strategic energy source, plays an extremely important role in optimizing energy structure and ensuring energy security. Fourth-generation nuclear energy has the outstanding advantages of high safety and efficiency, low investment, low waste generation, and a stronger nuclear non-proliferation capabilities through technological innovation, etc. It has revolutionary innovations compared with the previous three generations of nuclear power plants and is the only way to solve the contradiction between the continuous growth of energy and the greenhouse effect caused by the consumption of petrochemical fuels today [1,2,3,4]. The steam generator is the core equipment for the pressure boundary of the first/second loop of a nuclear power plant. It is not only the hub for efficient conversion of nuclear energy to electrical energy but also an important barrier to ensure the safe operation of the nuclear power plant and prevent the leakage of radioactive fission products. The heat transfer tube is a key component in the steam generator. The historical operating experience of nuclear reactors shows that more than half of the accidents with unit failure rates are caused by heat exchange tubes [5,6,7,8]. The increase in the total installed capacity of IV nuclear power systems and the improvement of unit parameters have put forward higher requirements for the materials of heat transfer tubes. Incoloy 800H is among the few structural alloys that are approved by the ASME Boiler & Pressure Vessel code. Due to its good thermal strength and corrosion resistance, it is considered a near-term candidate for heat exchange tubes in high-temperature gas-cooled reactors, supercritical water-cooled reactors, sodium-cooled fast reactors, and ultra-high-temperature reactors, which are the advanced reactor types of IV nuclear power systems [9,10,11]. The stable service of 800H is an important guarantee for the safe operation of nuclear power systems. Incoloy 800H alloy is developed by increasing the carbon content based on Incoloy 800, and it has superior properties compared to Incoloy 800 alloy. However, as an austenitic heat-resistant alloy, the manufacturing cost of 800H alloy is relatively high, and it has corrosion sensitivity within a certain temperature range, posing a significant threat to its safe and stable service [12,13].

Corrosion in the water chemical environment is an electrochemical process in essence. Revealing the corrosion mechanism of 800H is a key issue for its performance optimization and safe application. The intergranular corrosion, stress corrosion, and pitting corrosion of 800 alloy have received a fair amount of attention, and the electrochemical corrosion characteristics have been discussed [13,14,15]. The mechanical performance and oxidation behavior of 800H alloy have been investigated. It was reported that 800H showed an increase in strength but a decrease in ductility after steam corrosion testing at 575–650 °C up to 10,000 h (23.5 MPa) [16]. Further exposure at 900 °C/1000 h led to restoring the overall mechanical properties to levels close to the solution-annealed state [17]. Creep tests revealed that 800H demonstrated two main fracture mechanisms: necking and intergranular damage depending on loading conditions, and a higher Ti + Al content led to a lower strain rate and better creep resistance [18]. For the corrosion resistance performance, 800H showed obviously better resistance compared with AISI 304L SS, which is widely used as high-temperature construction material, and this was due to the continuous Cr_2_O_3_ that formed on the surface of 800H [19]. The environmental factors, such as the temperature, dissolved oxygen, pH, etc., were reported to exert significant impact. An increase in temperature and oxidation duration would obviously accelerate the oxidation of 800H; it is worth noting that oxidation intensified sharply at 900 °C, and the oxide film showed protrusions and discontinuity [19,20]. Increasing dissolved oxygen concentration also increased the corrosion rate obviously, while reliable oxide growth kinetics were not obtained due to the lack of data [21]. In impure helium atmospheres at 950 °C, different ratios of *P_H_*_2_/*P_H_*_2*O*_ and *P_CH_*_4_/*P_H_*_2*O*_ led to different oxidation and carburization behaviors of 800H alloy. When *P_H_*_2*O*_ was at a constant concentration, high *P_H_*_2_ reduced the oxidation capacity of the atmosphere, and high *P_CH_*_4_ caused severe carburization [22]. Study also revealed that the surface condition, i.e., surface grinding and cold rolling, may affect the corrosion resistance of 800H under the service conditions [23,24]. Su et al. [23] reported that the corrosion resistance of the ground specimens and heavily rolled specimens increased. However, contradictory research conclusions were also reported, i.e., cold work increased the corrosion rate of 800H and thus promoted selective oxidation and internal oxidation [24].

Microstructure quite often plays a vital role in controlling the corrosion behavior of materials. As a single-phase austenite alloy, solid solution treatment is the most important heat treatment of 800H for determining the subsequent processing properties, obtaining a uniform microstructure, and ensuring the final service performance of this material. Solid solution heat treatment can regulate the grain size, distribution of elements, second-phase particles or precipitates, grain boundary characteristics, etc., and thus the corrosion resistance. The dissolution and precipitation of second-phase particles or precipitates are highly dependent on the heat treatment temperatures. The effects of second-phase particles observed in 800H, such as M_23_C_6_ and TiN [25,26], on the corrosion resistance of 800H are still unclear. Temperature significantly affects the diffusion and distribution of elements, which vary significantly in their diffusion rate and solubility in the matrix. For example, carbon atoms diffuse much faster than other alloy elements in the material [14]. Different boundary types have vastly different energies and mobilities. Researchers reported that coherent Σ3 twins are highly resistant to sensitization and corrosion due to their ordered structure. However, incoherent Σ3 and other special boundaries (e.g., Σ7) can increase the corrosion sensitivity [27,28,29,30]. Among these, grain growth is the most obvious microstructural change during solid solution heat treatment. Grain size not only has a significant impact on the strength and toughness of alloy materials but also affects the chromium concentration gradient in the chromium-poor zone [31,32]. Thus, the grain size is monitored during the cold process and heat treatment cycle in its manufacturing process and is regarded as an important acceptance criterion for finished pipes. However, the relationship between solid solution heat treatment and grain size is not clear. Additionally, the corrosion behavior and mechanisms of 800H need clarification. As a corrosion-resistant alloy, the corrosion reactions during the corrosion process are still unclear, and the polarization characteristics have not been reported. Microstructure quite often plays a vital role in controlling the corrosion behavior of the materials, and studies interpreting the heat treatment–microstructure–corrosion relationship are essential to comprehend the failure mechanism and corrosion performance optimization of 800H.

This work investigated the effect of solid solution heat treatment on the corrosion resistance of 800H alloy using various electrochemical techniques. In addition, the corrosion mechanisms of the 800H alloy were analyzed and the microstructure effect was discussed. This work will contribute to filling the current corrosion research gap of 800H and promote its application in IV nuclear systems.

## 2. Materials and Methods

### 2.1. Materials and Samples

The 800H alloy used in this work was a hot-rolled billet from Baoyin Special Materials Technology Co., Ltd. (Yixing, China). Its chemical composition (wt.%) is as follows: Fe 43.27, Ni 32.21, Cr 22.43, Mn 0.77, Ti 0.52, Al 0.46, P 0.005, S 0.001, Si 0.18, C 0.091, Co 0.01, Cu 0.01, H 0.0001, and N 0.003. The billet was cut into small square samples with a size of 1 cm × 1 cm × 1 cm via wire electrical discharge machining (EDM) with the cutting parameters of 8 V, 3 A, and 60 mm^2^/min. The samples were ground, cleaned, and then dried with cold air for later use. The samples were heat treated at 1120 °C, 1150 °C, 1180 °C, and 1200 °C according to the ASTM standard [33] and previous research work. When the temperature of the furnace stabilized at the test temperature, the samples were introduced into the furnace for solid solution heat treatment for 12.5 min in the air atmosphere of the furnace chamber and then water quenched at the end.

After heat treatment, the samples were rinsed with ethanol and then deionized (DI) water. In order to observe the metallographic structure, they were manually abraded with silicon carbide paper from 300 grit up to 1500 grit and were then polished in a polish-grinding machine for a mirror surface. After a quick rinse by DI water, the polished samples were etched in etchant (20 mL HCl + 0.5 mL HNO_3_ + 5 mL FeCl_3_) for about 60 s and then used for optical and scanning electron microscope (SEM) metallographic analysis. The grain size distribution characteristics of 800H after different solid solution heat treatment were analyzed by Image J 1.52a. The total number of grains in the metallographic microscopy images for 1120 °C, 1150 °C, 1180 °C, and 1200 °C was 133, 95, 112, and 69, respectively.

For each heat treatment temperature, 3 samples were prepared for electrochemical testing. A copper wire was welded to one 1 cm × 1 cm face of each sample, and the sample was then mounted in epoxy resin with the other 1 cm^2^ surface area exposed. The exposed surface was abraded and polished to a mirror surface as described above, dried with cold air, and tested as soon as possible.

### 2.2. Experimental Procedures

Open circuit potential (OCP), linear polarization resistance (LPR), electrochemical impedance spectroscopy (EIS), and anodic potentiodynamic polarization were implemented to evaluate the corrosion behavior and electrochemical kinetics of the 800H alloy after different solid solution heat treatments in 3.5% NaCl solution. The OCP technique was applied to monitor the potential change in the 800H alloy sample until a stable potential, i.e., *E_corr_*, was obtained. The LPR technique was implemented from −0.015 to +0.015 V vs. *E_corr_* at a rate of 0.1 mV/s. Electrochemical impedance spectroscopy (EIS) was conducted based on the *E_corr_* value of the steel sample, with frequency varying from 100 kHz to 0.01 Hz and a sinusoidal potential signal with a maximum amplitude of 10 mV. Anodic potentiodynamic polarization was scanned from *E_corr_* to about 0.4 V vs. *E_corr_* at a scan rate of 0.5 mV/s. A three-electrode system was used for the test, where the 800H sample referred to above was used as the working electrode (WE); an Ag/AgCl electrode (potassium chloride [KCl] = 4 M) was used as a reference electrode (RE), and potential values are quoted with respect to this RE (0.197 V vs. SHE); a 2 cm × 1.5 cm platinum sheet was used as the counter electrode (CE).

The solution used for the electrochemical testing was 3.5% NaCl at room temperature. DI water and sodium chloride (NaCl, SCR, AR) were used to prepare the solutions. All of the electrochemical tests were operated by a CHI760E workstation (Shanghai CH Instruments Co., Ltd., Shanghai, China) and repeated three times separately. Surface characterization and scanning electron microscope metallographic analysis was conducted by an FEI NovoNano450 (Phenomworld, Eindhoven, The Netherlands). The optical metallographic microscope used was a Leica Dmi8C (Leica Microsystems CMS GmbH, Wetzlar, Germany). The heat treatment was conducted by Shanghai Siomm box-type furnace SXL-1400G (Shanghai Jvjing Precision instrucment Manufacturing Co., Ltd., Shanghai, China). The EDM machine was a Taizhou Wenjay DK7720 (Taizhou WenJay CNC Equipment Co., Ltd., Taizhou, China).

Intergranular corrosion tests were conducted according to GB/T 15260-2016 [34]. The samples after solid solution heat treatment at different temperatures were prepared according to the standard and then were exposed to boiling copper–copper sulfate–16% sulfuric acid solutions for 72 h. After testing, they were bent 180° over a diameter equal to the thickness of the specimen for visual examination as described by the standard, and the cross-sections of all samples were further analyzed by SEM.

## 3. Results

### 3.1. The Microstructure of the 800H Alloy After Different Solid Solution Heat Treatments

**Figure 1:**
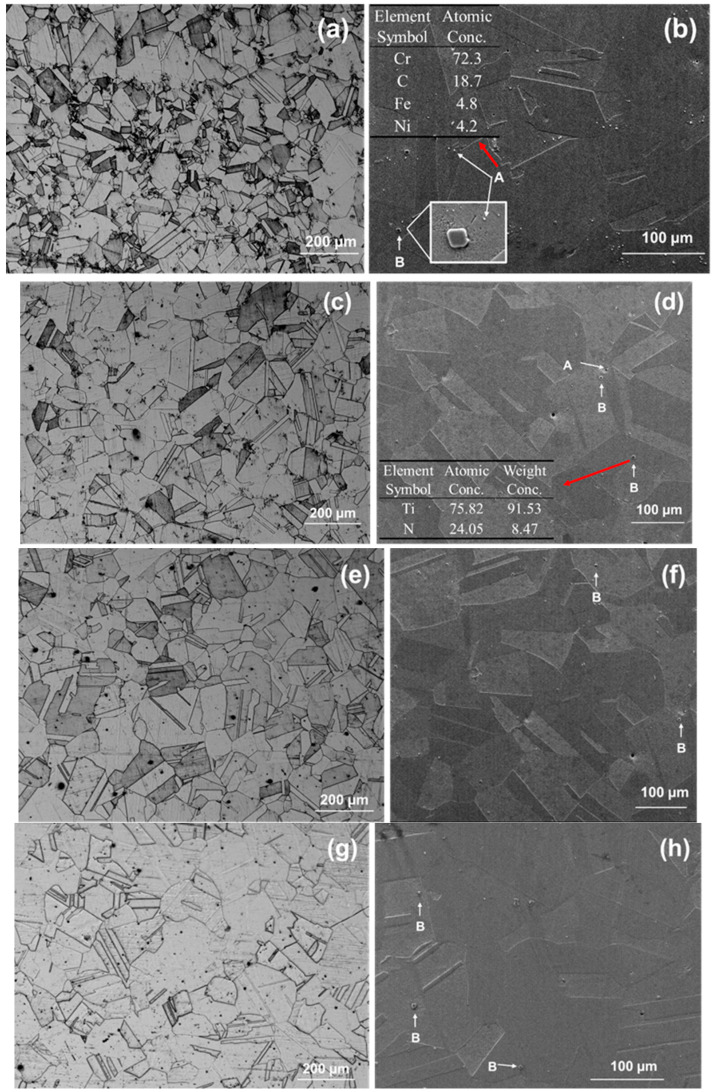
The metallographic pictures of different solid solution treatments: (**a**,**b**) 1120 °C; (**c**,**d**) 1150 °C; (**e**,**f**) 1180 °C; (**g**,**h**) 1200 °C. Images (**a**,**c**,**e**,**g**) are optical microscopy images, and images (**b**,**d**,**f**,**h**) are scanning electron microscopy images. Two types of second-phase particles, A and B were observed, and their EDS analysis is listed in (**b**) and (**d**), respectively.

Figure 1 shows the microstructure of 800H after solid solution treatment at 1120 °C, 1150 °C, 1180 °C, and 1200 °C. As a single-phase austenite structure, the 800H alloy did not involve phase transformation during high-temperature heat treatment, but the high-temperature heat treatment had a significant impact on its grain size, which was the most significant microstructural change. As shown in Figure 1, the grain size increased obviously with increasing temperature. At 1120 °C, the growth of grains was not uniform, and only some showed obvious enlargement, which indicated insufficient heat treatment. At 1150 °C, the grains grew uniformly with a good amount of twin boundaries and equiaxed grain structure. As the heat treatment temperature increased to 1180 °C and 1200 °C, the grain size significantly increased with straight grain boundaries. In addition, two types of second-phase particles were observed. Particles with a smaller size and an irregular shape were also observed, marked as A in Figure 1 for 800H after heat treatment at 1120 °C, and they are reported to be Cr-rich carbides with the form of M_23_C_6_ [11,12,35,36]. With the increase in solid solution temperature, the Cr-rich carbides generally dissolved due to the increased alloy element diffusion rate and solubility of carbides in the matrix, and the alloy became a homogeneous solid solution as the temperature increased to 1150 °C. In addition, particles with a regular shape, generally square or triangular, with a large size (1–5 um) were TiN, which were distributed within the grains or on the grain boundary, marked as B, and they were observed in all samples.

**Figure 2:**
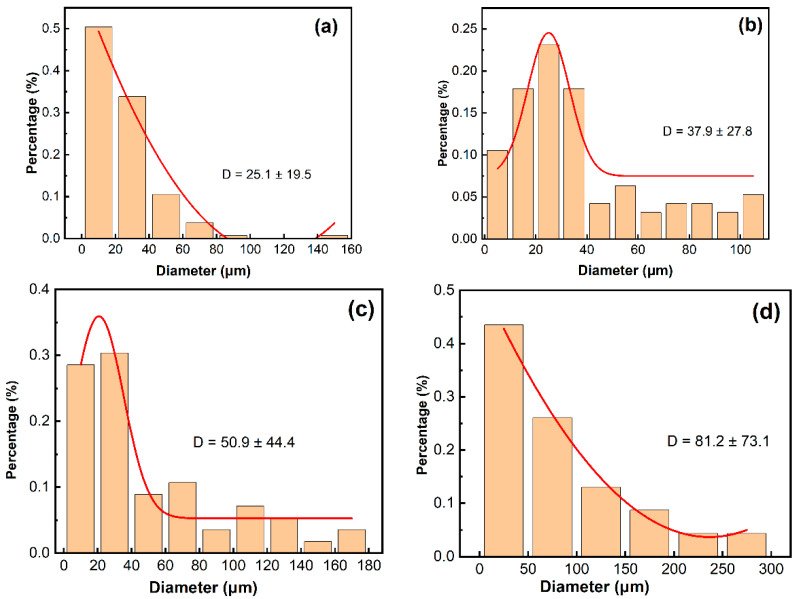
Grain size distribution statistics of 800H alloy after solid solution heat treatment at (**a**) 1120 °C; (**b**) 1150 °C; (**c**) 1180 °C; (**d**) 1200 °C.

The grain size distribution characteristics of 800H after different solid solution heat treatments are shown in Figure 2. As the solid solution heat treatment temperature increased from 1120 °C to 1150 °C, the average grain size increased from 25.1 µm to 37.9 µm, and the grain size showed a normal distribution with the best grain uniformity at 1150 °C. As the temperature increased to 1180 °C and 1200 °C, the grain size kept increasing with decreased uniformity. The average grain size reached 81.2 µm, and the largest grains reached 300 µm after heat treatment at 1200 °C. As reported by Chen et al. [37], the hardness of 800H decreased dramatically with deteriorated mechanical properties as the average grain size reached 60 µm. Thus, solid solution heat treatment at 1200 °C was not appropriate for 800H, and the corresponding corrosion behavior was not further investigated in this work.

The main driving force for grain growth is the decrease in total interfacial energy. Interface migration is accomplished by atoms overcoming certain barriers and can be regarded as a thermal activation process. This process can be described by the Arrhenius formula [38], as shown in the following formula:

(1) $$D^{2}-D_{0}^{2}=Aexp(-\frac{Q}{RT})$$

where *D* represents the average grain size at a certain solid solution temperature, m; *D*_0_ is the initial grain size, m; *A* is an influential factor, which is related to the nature of the material; *Q* represents the activation energy for grain growth, kJ/mol; *R* is the gas constant; *T* represents the solid solution temperature, K.

Considering $D^{2}\gg D_{0}^{2}$, Equation (1) can be simplified to Equation (2):

(2) $$D^{2}=Aexp(-\frac{Q}{RT}),i.e.,\;lnD=\frac{1}{2}lnA-\frac{Q}{2RT}$$

**Figure 3:**
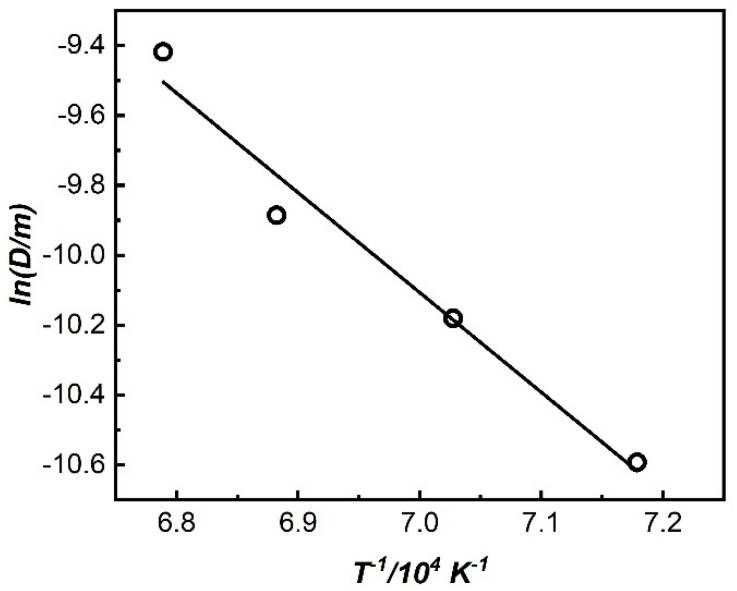
The relationship between *T*^−1^ and *lnD* based on the Arrhenius formula.

The graph of *T*^−1^-*lnD* is shown in Figure 3. It can be seen that the two have a good linear relationship. Based on the linear fitting analysis, the factor of *T*^−1^ can be calculated, and Equation (2) can be expressed as Equation (3); the grain growth activation energy of the 800H alloy subjected to the solid solution treatment is calculated to be 164.28 kJ/mol.

(3) $$lnD=\frac{1}{2}lnA-\frac{9.88}{T}$$

### 3.2. The Electrochemical Corrosion Behavior of the 800H Alloy After Different Solid Solution Heat Treatments

The classical Arrhenius model provides a fundamental baseline, but it is not the only one to govern grain growth in alloy systems like 800H. For real materials, it is an oversimplification where multiple competing mechanisms operate simultaneously. Factors that cause grain growth behavior in alloys to deviate from the simple Arrhenius relationship include (1) solute/impurity drag: solute atoms segregate to grain boundaries, pinning them and reducing mobility. This may create a non-linear relationship between mobility and temperature [39]; (2) second-phase particles: the second-phase particles or precipitates observed in 800H, such as M_23_C_6_ and TiN, could physically pin boundaries, halting or slowing the grain growth [25,26]; (3) grain boundary character and energy: different boundary types, e.g., Σ3 twin vs. random high-angle, have vastly different energies and mobilities, leading to non-uniform growth. In 800H, Σ3 boundaries have specific energies and behaviors that dominate microstructural evolution [30]; and (4) texture and processing history: deformation texture (from rolling) creates non-random grain orientations, favoring the growth of certain grains, leading to abnormal grain growth [40]. In 800H, cross-rolling induces microstructures prone to abnormal grain growth during annealing [41].

#### 3.2.1. Corrosion Potential and Linear Polarization Resistance Results

**Figure 4:**
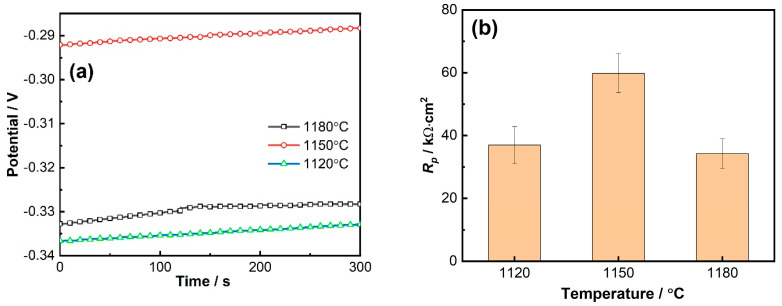
(**a**) Open circuit potential and (**b**) *R_p_* of 800H alloy after solid solution treatment at different temperatures.

The open circuit potential of the 800H alloy after solid solution heat treatments at 1120 °C, 1150 °C, and 1180 °C was monitored until a stable value, i.e., *E_corr_*, was obtained. The potential change during the last 5 min is shown in Figure 4a. The *E_corr_* was −0.335 V, −0.291 V, and −0.328 V, respectively, after solid solution treatment at 1120 °C, 1150 °C, and 1180 °C. The significantly high value of *E_corr_* at 1150 °C indicated a better corrosion resistance. The linear polarization resistance, i.e., *R_p_*, based on the linear polarization resistance measurement is shown in Figure 4b. The *R_p_* value of 800H heat treated at 1150 °C was 59.8 kΩ cm^2^, which was obviously higher than that obtained at 1120 °C (37 kΩ cm^2^) and 1180 °C (34.3 kΩ cm^2^). *R_p_* results were consistent with *E_corr_* results and indicated that the solid solution heat treatment temperature had an obvious effect on the corrosion resistance of 800H, and 800H after heat treatment at 1150 °C demonstrated the best corrosion resistance compared with that at 1120 °C and 1180 °C.

#### 3.2.2. EIS Results

**Figure 5:**
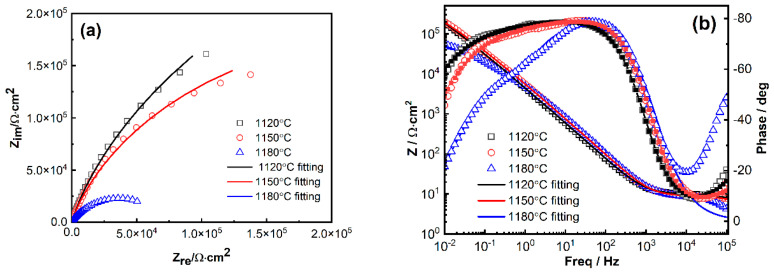
Electrochemical impedance spectroscopy (EIS) results of 800H alloy after solid solution treatment at different temperatures: (**a**) Bode plots and (**b**) Nyquist plots.

The surface corrosion product layer and interfacial phenomena of the 800H alloy were investigated by the EIS method. Impedance spectra for the 800H alloy after solid solution heat treatment at 1120 °C, 1150 °C, and 1180 °C are shown as Bode plots (Figure 5a) and Nyquist plots (Figure 5b). All of the 800H alloy samples after heat treatment at different temperatures demonstrated a highly capacitive response in the Nyquist plot (Figure 5a) and approached large phase angles over a relatively wide frequency range (from 10^−1^−10^1^ Hz). The impedance magnitude of |Z| was large at the low frequency limit. These results suggested that a layer of protective and compact corrosion product may form on the surface of the samples. Detailed characterization of this film is worthy in future work [11,12,13,14]. It was noted that the 800H sample heat treated at 1180 °C showed a relatively narrow phase angle range and small |Z| at the low frequency limit, and this indicated a relatively poor protective performance of the corrosion product film that formed on its surface.

**Figure 6:**
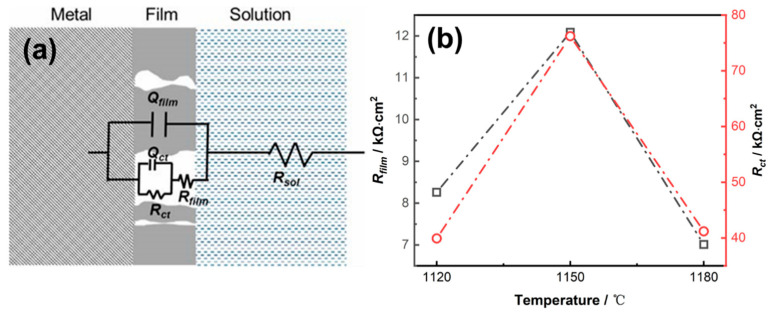
(**a**) The two-time constant electrical equivalent circuit (EEC) used to fit the EIS spectra, (**b**) the fitting results of film resistance (*R_film_*) and charge transfer resistance (*R_ct_*) for 800H alloy after solution heat treatments at different temperatures.

In order to further interpret the interfacial characteristics, a two time-constant electrical equivalent circuit (EEC), *R_sol_*(*Q_film_*(*R_film_*(*Q_i_R_ct_*))), is used to interpret the EIS plots (Figure 6a). In this EEC, *R_sol_* is the solution resistance; *R_film_* is the surface product film resistance; *Q_film_* corresponds to the capacitive behavior of the product film; and *R_ct_* and *Q_i_* account for the charge transfer resistance and the capacitance of the electrical double layer, respectively. The constant phase element, *Q*, instead of a capacitor *C*, was adopted here to compensate for the non-ideal capacitive behavior of the surface and the distribution of relaxation times resulting from different degrees of heterogeneities at the electrode surface [41,42]. The fitting was conducted in ZsimpWin-3.30d software, and the fitting quality was evaluated by the chi-squared (χ^2^) values. The fitting results are plotted along with the EIS data in Figure 4 and also shown in Table 1. As shown in Figure 5, the two-time constant EEC gives a near-perfect fit to the measured data.

The product film resistance, *R_film_*, represents the corrosion resistance of the passive metals to a large extent, and its fitting results are illustrated in Figure 5b. The considerable values of *R_film_* indicated the presence of product film. As noted, *R_film_* increased as the solid solution heat treatment temperature increased from 1120 °C to 1150 °C but decreased as the temperature increased further to 1180 °C. The *R_ct_* value of the 800H alloy after different heat treatments was also included in Figure 6b. The large value of *R_ct_* at 1150 °C suggested the retarded reactions and better corrosion resistance of 800H.

#### 3.2.3. Anodic Polarization Curves

**Figure 7:**
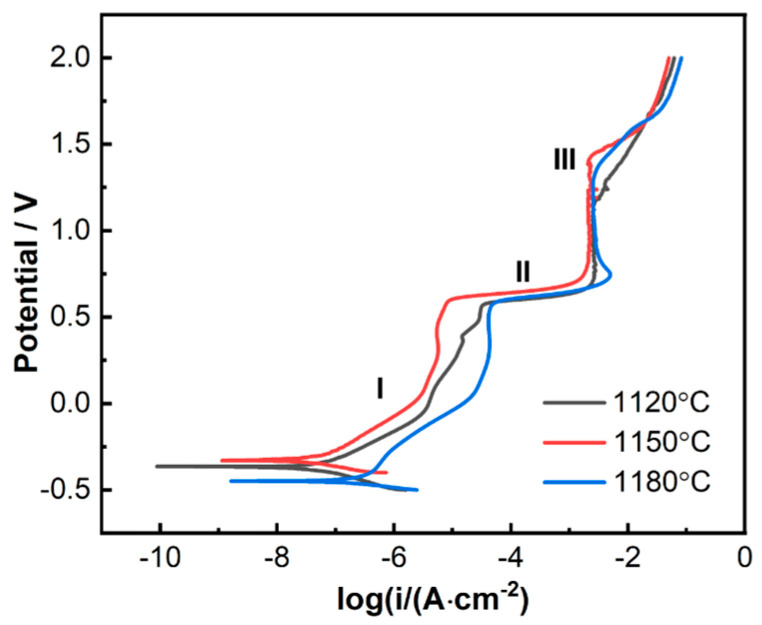
The anodic polarization curves of 800H alloys after different heat treatments in 3.5% NaCl solutions at room temperature.

The anodic polarization curves of 800H are shown in Figure 7. All three 800H alloy samples with different microstructures after heat treatments showed similar polarization characteristics in general: pseudo-passivation (marked as I in the plot), transpassivation (II), and repassivation (III) with an increase in potential. In the first stage (I), the anodic polarization current densities of the 800H alloy samples increased slightly with the positive shift of potential. This pseudo-passivation behavior indicated the formation of a relatively protective passive film on the surface, which inhibited the rapid corrosion of the 800H alloy. At this stage, the 800H substrate oxidized and accounted for the polarization current.

800H is mainly composed of Fe, Ni, and Cr elements. In this low-polarization region, the anodic oxidation reactions were dominated by the oxidation of Cr and Fe (as shown in Equations (4) and (8)), and the oxidation of metal Ni occurred later, as shown in Equation (9):

Cr → Cr^2+^ + 2e^−^ (4)

Cr → Cr^3+^ + 3e^−^ (5)

Cr^2+^ → Cr^3+^ + e^−^ (6)

Fe → Fe^2+^ + 2e^−^ (7)

Fe → Fe^3+^ + 3e^−^ (8)

Ni → Ni^2+^ + 2e^−^ (9)

In the second stage (II), the polarization current increased dramatically as the potential reached 0.55 V, and this transpassivation behavior suggested the breakdown of the previous passivation film and fast corrosion rate of the 800H alloy. Based on the equilibrium electrode potentials, the dominant reaction that accounted for the obvious current increase at 0.56 V is shown in Equation 10, where the low-valent Ni was oxidized to form higher-valent nickel:

Ni(OH)_2_ + OH^−^ → NiOOH + H_2_O + e^−^ (10)

With a further increase in potential, the 800H alloy was rapidly passivated and maintained almost a constant passive current for a large potential range, as shown in the third stage (III). The dominant reaction that resulted in this passivation is shown as Equation (11):

2FeO + 2OH^−^ → Fe_2_O_3_ + H_2_O+ 2e^−^ (11)

Due to the formation of Fe_2_O_3_, etc., on the surface of the sample, the oxide film became compact and protective, and it effectively hindered the mass transfer process of the corrosion reactions. Therefore, the anodic polarization current did not increase obviously with the increase in potential, remaining in a stable passivation region. When the potential increased further up to 1.2 V, the electrolytic reaction of water occurred and was attributed to the increased polarization current densities, as shown in Equation (12):

2H_2_O → O_2_ + 4H^+^ +4e^−^ (12)

It is worth noting that the anodic polarization current densities of the 800H alloy after heat treatment at 1150 °C were slightly smaller compared with the other two samples heat treated at 1120 °C and 1180 °C. This indicated a better corrosion resistance of the 800H alloy after heat treatment at 1150 °C.

**Figure 8:**
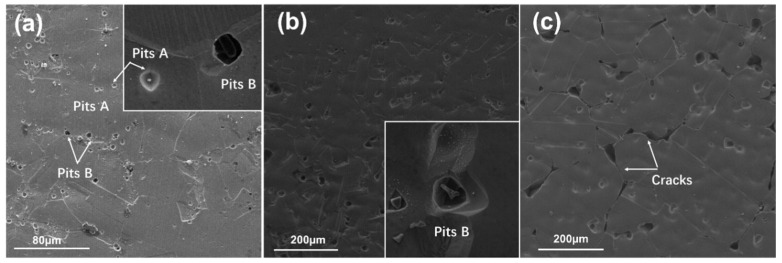
The surface morphology after anodic polarization test: (**a**) 1120 °C, (**b**) 1150 °C, (**c**) 1180 °C. Corrosion pits A corresponded to the second phase of M_23_C. Corrosion pits B corresponded to TiN inclusions.

The surface morphology of 800H after anodic polarization testing is shown in Figure 8. Corrosion pits with different sizes were present on the samples. Corrosion pits A, as shown in Figure 8 corresponding to the second phase of M_23_C, were relatively small and shallow. Corrosion pits B corresponded to TiN inclusions and were big and deep. The surface morphologies suggested that the second-phase particles acted as active sites and resulted in the corrosion that preferably initiated around them, and these results are consistent with those reported by other researchers [22,35]. It was noticed that in Figure 8c for the 800H after heat treatment at 1180 °C, evident corrosion pits and cracks along the grain boundaries were observed, e.g., intergranular corrosion (IGC), as marked in the picture. This suggested that the grain boundaries were the dominant place for anodic polarization reactions, and it is also the weak area subjected to corrosion attack.

**Figure 9:**
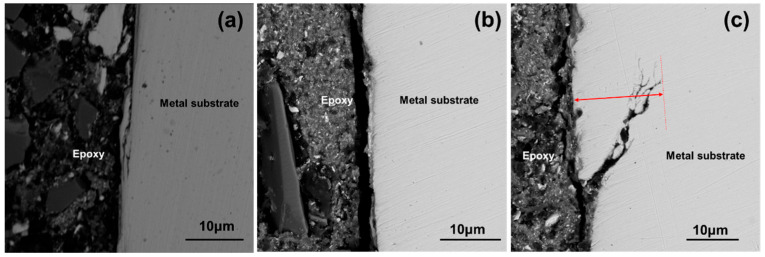
Intergranular corrosion of 800H after solid solution heat treatment at (**a**) 1120 °C; (**b**) 1150 °C; (**c**) 1180 °C, the red arrow demonstrated the depth of IGC.

In order to further verify the IGC of 800H after different heat treatments, intergranular corrosion tests were conducted. No obvious cracks were observed on all samples after bending based on a visual examination. The SEM photos of their cross-sections after testing are shown in Figure 8. As indicated by the arrow in Figure 9, no obvious IGC cracks were observed on the 800H alloy after heat treatment at 1120 °C and 1150 °C. As the solid solution treatment temperature was increased to 1180 °C, IGC cracks as deep as 17 μm were observed. The IGC tests suggested that the 800H alloy with a large grain size demonstrated high IGC sensitivity after high temperature solid solution heat treatment.

## 4. Discussion

Solid solution heat treatment is the most important treatment for 800H. The results from this work revealed that solid solution heat treatments significantly affected the microstructure of 800H and thus the corrosion-resistant performance.

### 4.1. Corrosion Initiated by the Second Phase

Carbides were widely present in the matrix after solid solution heat treatment at 1120 °C. Due to the uneven structure and electrochemical activity, a galvanic-type corrosion cell formed between the carbides and the matrix. The carbides were relatively inert in comparison with the matrix, and they acted as cathodes and accelerated the dissolution of the surrounding matrix in the micro-corrosion couples [22,35]. Thus, corrosion pitting occurred on the matrix side of the interface between the inclusions and the substrate, which were commonly observed on 800H heat treated at 1120 °C.

Another important cause is TiN. The 800H used in this work contains 0.52% Ti and 0.003% N. The addition of Ti could refine the grains and form strong nitrogen and/or carbon compounds, thus decreasing the IGC sensitivity. Because of the extra-low formation free energy of TiN, the primary formation of TiN is unavoidable during the solidification process [28]. As indicated in Figure 7, the junction of Ti-containing inclusions and the substrate is also the location where corrosion occurs preferentially due to the galvanic couple cell effect. In addition, there is a significant mismatch between TiN inclusions and the austenite matrix; thus, the local concentrated stress tended to cause corrosion initiation and cracking on the interface between the inclusions and matrix [43,44]. Lastly, as the large TiN particles located on the grain boundaries fell off while galvanically coupled with the matrix, a local stress concentration occurred at their original locations, and this can further promote the corrosion propagation. The preferential corrosion attack around TiN is also emphasized by Li et al. [43] and Dutta et al. [44] in their research on the corrosion behavior of 690 alloy.

### 4.2. Intergranular Corrosion Test Results

800H showed apparent corrosion grooves along the grain boundaries after solid solution heat treatment at 1180 °C. The chemical distribution, grain boundary types, grain size, etc., were all reported to exert a significant effect on the intergranular stress corrosion resistance. Researchers widely believe that the chemical distribution, especially chromium distribution, is of vital importance, and a high equilibrium chromium concentration at the intergranular carbide-matrix interface is beneficial for IGC resistance [45,46]. Wang et al. [14] stated that carbon atoms diffuse much faster than other alloy elements in the material, and carbon activity is assumed to be uniform throughout the whole matrix. During high-temperature heat treatment, carbides may form at the grain boundaries. Because chromium atoms are relatively strong carbide formers, carbide formation decreased the interfacial chromium concentration and left a steep chromium concentration gradient. This may explain the increased IGC of 800H at 1180 °C [14]. For the grain boundary types, Zeng et al. [31] proposed that low-angle grain boundaries can effectively deflect IGC cracks into grains with lower corrosion susceptibility based on a phase-field model. Twin boundaries may mitigate corrosion crack propagation by reducing potential initiation sites and inhibit chromium depletion [31,47]. Tokita et al. [48] optimized grain boundary character distributions by thermomechanical processing and found the samples with over 80% frequency for coincident site lattice boundaries and disconnected random boundaries exhibited excellent IGC resistance. For the effect of grain sizes, however, there exist many contradictory results on the corrosion resistance. Due to the different grain refining approaches adopted, many interference factors would be introduced other than grain size, such as the surface residual stress, inclusion refinement, crystallographic defects, etc. Thus, studies claiming that grain refinement can lead to better corrosion resistance usually adopted grain-refining approaches that resulted in rather non-equilibrium microstructures, while the fully recrystallized grains showed the opposite tendency [32]. Comprehensive analysis of the elemental distribution, grain boundary characteristics, grain size, etc., and their synergistic effect on IGC resistance are necessary for a deep understanding of IGC. In addition, an investigation of the interfacial/microstructural analysis pertaining to the passive film that formed on the surface is also conducive to elucidate the underlying mechanisms. The primary objective of this work is to reveal the relationship between the microstructure-corrosion resistance of 800H. The related work, while valuable, is beyond its immediate aims and will be considered as the future plan.

**Figure 10:**
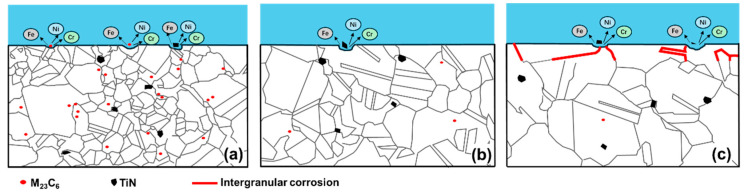
Microstructure evolution and corrosion mechanism of 800H after solid solution heat treatment at (**a**) 1120 °C, where grains grew unevenly and corrosion initiated at Cr-carbides and TiN inclusions; (**b**) 1150 °C, where grains grew evenly with the best grain uniformity, and corrosion initiated at TiN inclusions; and (**c**) 1180 °C, where intergranular corrosion was the main failure form.

Based on this work, the relationship between solid solution heat treatment, microstructure corrosion resistance, and the corrosion mechanism of 800H can be illustrated as Figure 10. Solid solution at 1120 °C was insufficient heat treatment and resulted in the uneven growth of grains. In addition, the second phases, especially the Cr-carbides, were not fully dissolved in the matrix. The corrosion mechanism for this case can be illustrated as Figure 10a. The widely distributed Cr-carbides and a relatively small number of TiN inclusions acted as cathodes and formed numerous galvanic-type corrosion cells within the matrix, and corrosion would initiate preferably at these second-phase particles. Thus, a large amount of corrosion pits would occur. When the solid solution temperature was 1150 °C, grains grew evenly with the best grain uniformity, and 800H exhibited the maximum *R_film_* and *R_ct_* values and minimum current densities. In addition, a good amount of twin boundaries occurred, and Cr-carbides dissolved into the matrix. Corrosion was mainly initiated by TiN inclusions and was significantly inhibited (as shown in Figure 10b). When the solid solution heat treatment temperature was increased to 1180 °C and above, grain size significantly increased with straight grain boundaries. In addition to the corrosion pits initiated by TiN inclusions, IGC was the main failure form, as illustrated in Figure 10c.

## 5. Conclusions

Solid solution heat treatment is the most important process for the manufacturing and final service performance of 800H alloy pipes. This work investigated the effect of heat treatment temperature on the microstructure of an 800H alloy and thus its corrosion resistance. The main conclusions are shown below:

- Solid solution at 1120 °C was insufficient heat treatment. The 800H alloy showed uneven growth of grains and undissolved Cr-carbides. Solid solution at 1150 °C resulted in even growth of grains with the best grain uniformity. Cr-carbides dissolved into the matrix and a good amount of twin boundaries was observed. Solid solution at 1180 °C and above resulted in overheating of the 800H alloy, and grain size significantly increased with straight grain boundaries.
- Electrochemical corrosion tests demonstrated that the 800H alloy exhibited the best corrosion resistance after heat treatment at 1150 °C. The 800H alloy showed pseudo-passivation, transpassivation, and repassivation (III) during the anodic polarization. This corresponded to the multi-reactions involved in the corrosion process.
- For 800H heat treated at 1120 °C, the widely distributed Cr-carbides and TiN inclusions formed galvanic-type corrosion cells within the matrix and resulted in corrosion pit initiation. In comparison, 800H heat treated at 1150 °C demonstrated less corrosion attack due to the dissolved Cr-carbides. However, as the solid solution temperature increased to 1180 °C, IGC sensitivity increased and IGC became the dominant failure form.

## Figures and Tables

**Table 1:** Electrical circuit parameter values for EECs obtained by fitting EIS spectra in Figure 3 and Figure 4.

Temperature/ °C	*R_sol_* /Ω·cm^2^	*Q_film_*-Y_0_ S·s^n^/cm^−2^	*Q_film_*-n (0 < n < 1)	*R_film_* /kΩ·cm^2^	*Q_i_*-Y_0_ S·s^n^/cm^−2^	*Q_i_*-n (0 < n < 1)	*R_ct_* /kΩ·cm^2^	χ^2^
1120 °C	9.371	5.139 × 10^−5^	0.8457	8.26	4.911 × 10^−5^	0.6311	39.9	0.00602
1150 °C	10	3.173 × 10^−5^	0.646	12.09	2.106 × 10^−5^	0.9338	76.2	0.03967
1180 °C	7.723	3.112 × 10^−5^	0.8902	7.01	5.076 × 10^−5^	0.6937	41.2	0.00884

## Data Availability

The original contributions presented in this study are included in the article. Further inquiries can be directed to the corresponding authors.

## Abbreviations

The following abbreviations are used in this manuscript:

*E_corr_*	Corrosion potential
OCP	Open circuit potential
*R_p_*	Linear polarization resistance
EIS	Electrochemical impedance spectroscopy
